# Dual loss of human *POLQ* and *LIG4* abolishes random integration

**DOI:** 10.1038/ncomms16112

**Published:** 2017-07-11

**Authors:** Shinta Saito, Ryo Maeda, Noritaka Adachi

**Affiliations:** 1 Department of Life and Environmental System Science, Graduate School of Nanobioscience, Yokohama City University, Yokohama 236-0027, Japan; 2 Department of Biology, Graduate School of Science, Chiba University, Chiba 263-8522, Japan; 3 Advanced Medical Research Center, Yokohama City University, Yokohama 236-0004, Japan

## Abstract

Homologous recombination-mediated gene targeting has greatly contributed to genetic analysis in a wide range of species, but is highly inefficient in human cells because of overwhelmingly frequent random integration events, whose molecular mechanism remains elusive. Here we show that DNA polymerase θ, despite its minor role in chromosomal DNA repair, substantially contributes to random integration, and that cells lacking both DNA polymerase θ and DNA ligase IV, which is essential for non-homologous end joining (NHEJ), exhibit 100% efficiency of spontaneous gene targeting by virtue of undetectable levels of random integration. Thus, DNA polymerase θ-mediated end joining is the sole homology-independent repair route in the absence of NHEJ and, intriguingly, their combined absence reveals rare *Alu*-*Alu* recombination events utilizing a stretch of homology. Our findings provide new insights into the mechanics of foreign DNA integration and the role of DNA polymerase θ in human genome maintenance.

## Abstract (web summary)

Homologous recombination mediated gene targeting is highly inefficient in human cells due to random integration events, Here the authors show that dual repression of polymerase θ and DNA ligase IV eliminate random integration events.

Since the first reports in the mid-1980s (refs 1, 2), efficient gene targeting has been hampered by random integration (RI), which typically occurs several orders of magnitude more frequently than homologous recombination (HR)-mediated targeted integration^3^. In several fungi, inactivation of the non-homologous end joining (NHEJ) pathway results in ∼100% gene targeting by virtue of undetectable levels of RI^4^, but unfortunately this is not the case in mammalian cells; for example, loss of DNA ligase IV (Lig4), which is indispensable for NHEJ^5^, can reduce RI of foreign DNA, but does not reduce RI of targeting vectors harbouring long homology arms^6^,^7^,^8^. Thus, the exact contribution of NHEJ to RI is still uncertain and undoubtedly an NHEJ-independent route for RI does exist.

NHEJ and HR are the two major pathways for repairing DNA double-strand breaks (DSBs)^9^,^10^. NHEJ absolutely requires the Ku70–Ku80 complex and the Xrcc4 (X-ray repair cross-complementing 4)–Lig4 complex to fix DSBs, with the aid of additional factors such as DNA-dependent protein kinase catalytic subunit (DNA-PKcs), Artemis, DNA polymerases μ and λ, XLF (Xrcc4-like factor)/Cernunnos and PAXX (paralogue of Xrcc4 and XLF) when the DSB is not readily ligatable^11^,^12^,^13^. NHEJ-mediated repair is more error-prone than HR, because it accompanies frequent nucleotide loss and addition^5^. In addition to NHEJ, mammalian cells possess at least one genetically distinct pathway for homology-independent repair termed alternative end-joining (A-EJ)^14^,^15^, which is considered suppressed by NHEJ and HR^16^,^17^,^18^. DSB repair by A-EJ is even more inaccurate than NHEJ, as it typically involves end resection of the DNA ends that is reliant on carboxy-terminal binding protein interacting protein (CtIP) and the Mre11 (Meiotic recombination 11)-Rad50-Nbs1 (Nijmegen breakage syndrome protein 1) complex^19^,^20^, although this end resection is not as extensive as that required for initiating HR or single-strand annealing (SSA)^13^,^21^. SSA represents another homology-dependent repair^22^, which is potentially deleterious to genome integrity, particularly when the genome contains many repetitive DNA sequences, as exemplified by *Alu* elements that are present at >1 million copies in the human genome and comprise ∼11% of human DNA^23^,^24^.

Although the exact molecular mechanism of A-EJ is still less characterized, accumulating evidence implicates DNA polymerase θ (Pol θ) in A-EJ^25^,^26^,^27^. Pol θ is a proofreading-deficient A-family DNA polymerase and is encoded by the *POLQ* gene^28^,^29^,^30^. Pol θ is unique among DNA polymerases as it possesses a helicase-like domain at its amino terminus^31^. Several recent studies have shown that loss of Pol θ results in decreased DSB repair activity^32^,^33^,^34^; however, it remains an open question whether Pol θ explains all non-homologous DSB repair other than NHEJ. Also unexplored is whether Pol θ contributes to RI of transfected DNA.

In this study, we show that human Pol θ is indispensable for A-EJ, which is the sole homology-independent DSB repair route in the absence of NHEJ and is typically characterized by either 2–6 bp microhomology or templated insertions at the junctions. Remarkably, despite its minor role in chromosomal DSB repair, Pol θ substantially contributes to RI and cells doubly deficient in Pol θ and Lig4 exhibit 100% gene-targeting efficiency because of virtually no RI events. Further, we provide the first direct evidence that the dual loss of Pol θ and Lig4 permits, albeit infrequent, SSA-type recombination between two separate *Alu* elements. Our findings provide new insights into the mechanics of foreign DNA integration and the role of Pol θ in preserving genome integrity.

## Results

### Characterization of Lig4-independent RI events

**Figure 1:**
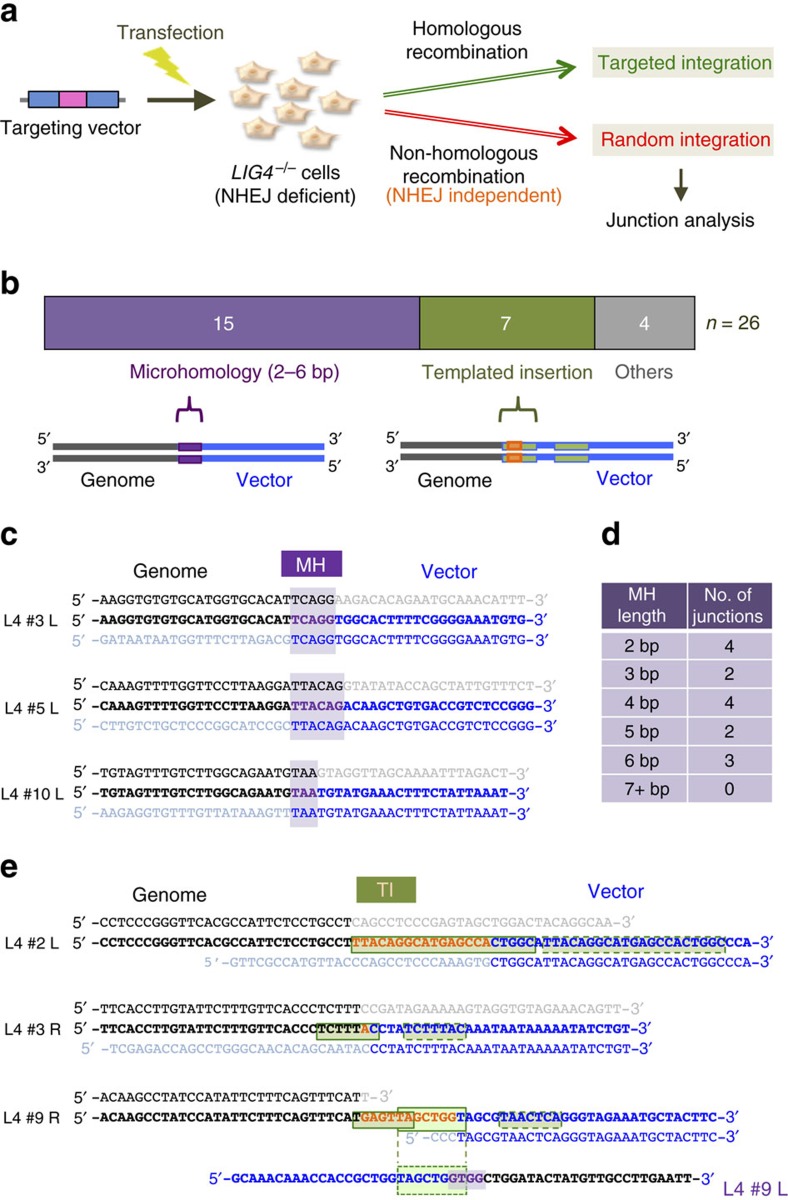
Characterization of Lig4-independent RI events. (**a**) Scheme for analysis of Lig4-independent RI. A promoterless targeting vector, p4.5 HPRT-2A-EGFP-2A-Puro, was transfected into human *LIG4*-knockout cells and the junctions of RI clones were analysed (see Supplementary Fig. 1). (**b**) Features and distribution of Lig4-independent junctions. The features were classified into three categories; microhomology (2–6 bp homology), templated insertion (≥6 bp direct or inverted repeats) and others showing 1–10 bp of undefined insertions. (**c**) Representative junction sequences relying on microhomology. Shown are the 5′ (left) junctions of clones #3, #5 and #10 (see Supplementary Data 1). Genome and vector sequences deleted after the RI event are shown in grey and faint blue, respectively. (**d**) Distribution of lengths of microhomology observed in the 15 junctions. (**e**) Representative junction sequences accompanying templated insertions. Shown are the 5′-junction of clone #2 and the 3′-junctions of clones #3 and #9 (see Supplementary Data 1). Inserted nucleotides are indicated in orange. It is noteworthy that the sequence surrounded by a square is identical to a nearby sequence surrounded by a dotted-line square or, as can been seen at the 3′- (right) junction of clone #9 (bottom), to a sequence present in the other (left) side of the junctions (see Supplementary Data 1).

To investigate the mechanism of Lig4-independent RI, we used a promoterless targeting vector, p4.5 HPRT-2A-EGFP-2A-Puro^35^, to perform gene targeting in *LIG4*-knockout cells derived from a human diploid cell line, Nalm-6 (refs 36, 37), and sought to characterize the nature of random integrants (‘RI clones’) arising in an NHEJ-independent manner (Fig. 1a and Supplementary Fig. 1). Sequence analysis of vector-genome junctions of those RI events revealed that 15 out of 26 (58%) utilized 2–6 bp microhomology for joining with no insertions (Fig. 1b–d and Supplementary Data 1). The remaining 11 junctions were associated with insertions, 7 of which were characterized by inclusion of a unique feature called ‘templated insertions’^25^,^26^, whereas 4 junctions contained 1–10 bp undefined insertions (Fig. 1b). Consistent with the intrinsic complexity of RI events^38^, the precise mechanisms for several junctions associated with templated insertions were difficult to interpret (Supplementary Data 1), but some junctions showed a simple pattern of templated insertions (that is, ≥6-bp direct or inverted repeats), including those copied sequences from the other side of the vector-genome junctions (Fig. 1e). As the traits of the templated insertions resemble those of Pol θ-mediated end joining recently reported in non-mammalian species^39^,^40^, we reasoned that Pol θ should be involved in at least some, and perhaps most, of RI events occurring in *LIG4*-knockout cells.

### Loss of Pol θ and Lig4 eliminates RI

**Figure 2:**
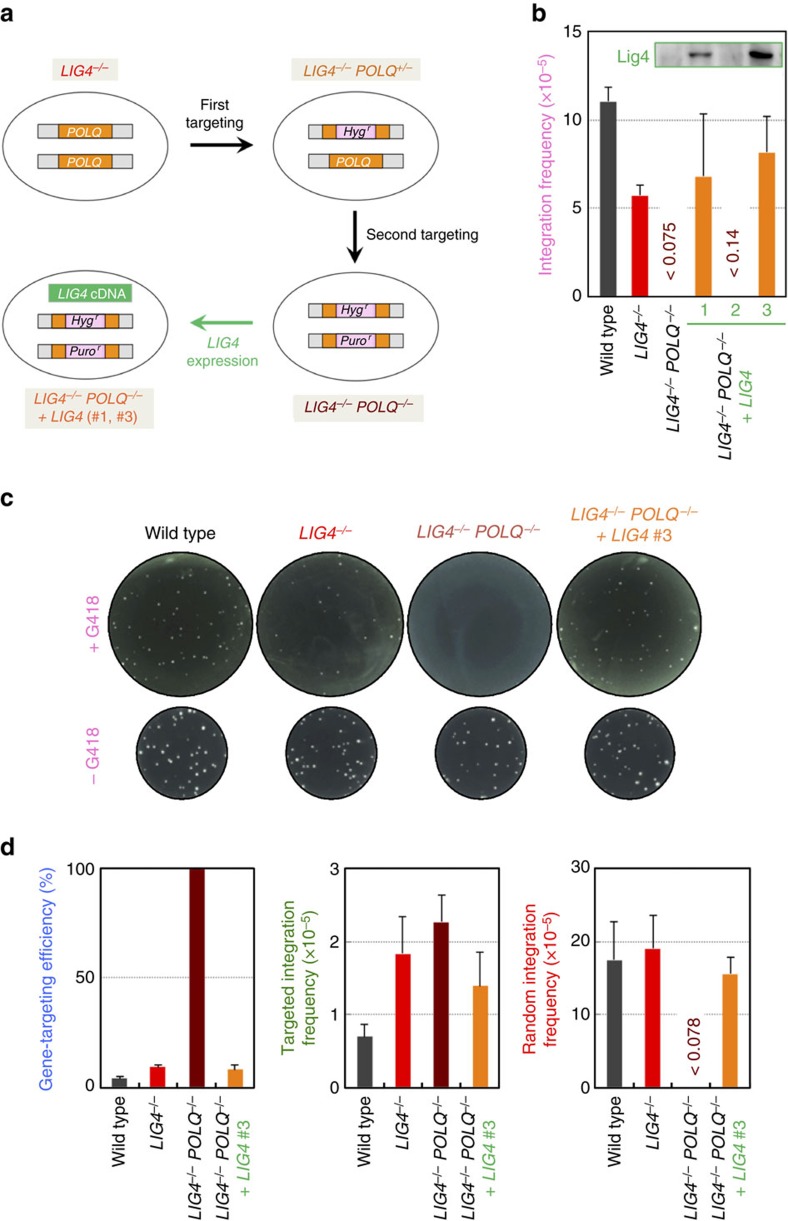
Dual loss of Pol θ and Lig4 suppresses RI. (**a**) Scheme for construction of mutant cell lines by gene targeting. The two *POLQ* alleles of *LIG4*-knockout cells were sequentially disrupted by HR-mediated gene targeting to yield heterozygous (*LIG4*^*−/−*^*POLQ*^*+/−*^) and homozygous (*LIG4*^*−/−*^*POLQ*^*−/−*^) knockout cells. Ectopic expression of *LIG4* cDNA in *LIG4*^*−/−*^*POLQ*^*−/−*^ cells yielded two *LIG4*-complemented cell lines (#1, #3). It is noteworthy that clone #2 failed to express Lig4 (see the inset in **b**) and thus served as a negative control for further analysis. (**b**) Integration frequency of pPGKneo in wild-type, *LIG4*^*−/−*^, *LIG4*^*−/−*^*POLQ*^*−/−*^ and *LIG4*-complemented *LIG4*^*−/−*^*POLQ*^*−/−*^ cells (mean±s.d.; *n*=3). Inset: western blot analysis for Lig4 (extracted from Supplementary Fig. 2f; shown for reference only). (**c**) Representative colonies arose in an experiment of **b**. Digital photographs were taken after three weeks of colony formation. Upper: G418-containing plates. Lower: G418-free plates. (**d**) Gene-targeting efficiency, targeted integration frequency and RI frequency of pHPRT8.9-Neo2(−) in wild-type, *LIG4*^*−/−*^, *LIG4*^*−/−*^*POLQ*^*−/−*^ and *LIG4*-complemented *LIG4*^*−/−*^*POLQ*^*−/−*^ cells (mean±s.d.; *n*=3).

To investigate the involvement of Pol θ in RI, we knocked out the *POLQ* gene (which encodes Pol θ) by gene targeting in *LIG4*-knockout cells (Fig. 2a and Supplementary Fig. 2a–e). The double-knockout cells grew slower, but were viable (Supplementary Fig. 2k) and showed wild-type levels of transfection efficiency (Supplementary Fig. 2l,m), thus allowing us to perform RI assay (Supplementary Fig. 3a). Astonishingly, pPGKneo-electroporated double-knockout cells gave rise to no G418-resistant colonies (Fig. 2b,c). We consistently obtained the same results when we repeated RI assay more than ten times by treating a larger number of cells (0.8 × 10^8^ in total) (Fig. 2b and Supplementary Fig. 3b) or by employing a Nucleofector technology for transfection (Supplementary Fig. 3c). By contrast, double-knockout cells complemented with *LIG4* cDNA were proficient in producing RI clones (Fig. 2b,c and Supplementary Figs 2f and 3c). In addition, very similar results were obtained using independently constructed *POLQ*-knockout Nalm-6 cells (Supplementary Fig. 3d).

We next performed gene-targeting assays at the *HPRT* locus using a promoter-containing targeting vector, pHPRT8.9-Neo2(−) (Supplementary Fig. 3e), which normally gives rise to a considerable amount of RI clones. Given the absence of RI events, we anticipated that all the drug-resistant colonies derived from double-knockout cells would be targeted clones and this was indeed the case (Fig. 2d). The 100% efficiency of gene targeting was consistently observed at several other loci such as the *AAVS1* safe-harbour locus, irrespective of homology-arm length or marker-gene cassette of the targeting vector (Table 1). The *LIG4*-complemented double-knockout cells as well as *POLQ*-knockout cells did not exhibit ultrahigh targeting efficiency (Fig. 2d and Supplementary Fig. 3f–h), demonstrating that the presence of either Pol θ or Lig4 is sufficient to bring about RI.

### Evidence for Pol θ-mediated RI in NHEJ-proficient cells

**Figure 3:**
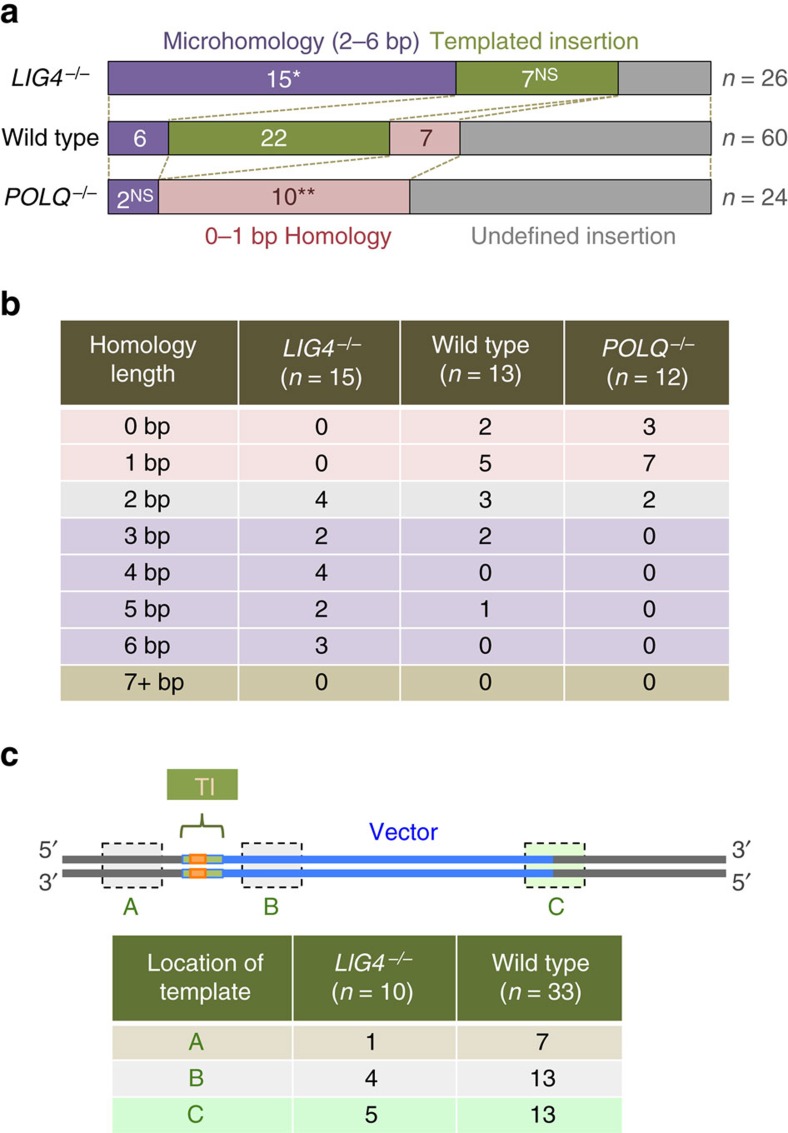
Evidence for Pol θ-mediated RI in NHEJ-proficient cells. (**a**) Features and distribution of junctions in RI clones from wild-type and *POLQ*^*−/−*^ cells. The junction sequences are shown in Supplementary Data 2 and 3. The features were classified into four categories; microhomology (2–6 bp homology), templated insertion (≥6 bp direct or inverted repeats), undefined insertion (1–11 bp) and 0–1 bp homology. The *LIG4*^*−/−*^ data (same as Fig. 1b) is shown for reference only. A Fisher’s exact test was used to determine statistical significance. NS, not significant; *P<0.00001 and **P<0.01. Although not indicated, the absence of templated insertion in *POLQ*^*−/−*^ cells is statistically significant (*P*<0.0003). (**b**) Distribution of junctions that arose from direct (insertion-free) end joining in terms of terminal homology length. The *LIG4*^*−/−*^ data is the same as Fig. 1d. (**c**) Distribution of templated insertion events in terms of location of the original template sequence. The locations were categorized into three groups: a genome sequence present near the junction (A), a vector sequence present near the junction (B) and a sequence present at or near the other (opposite) junction of the integration site (C); although not shown in the figure, a vector sequence deleted after RI can also be used as a template. It is noteworthy that *POLQ*^*−/−*^ data are not presented owing to the complete absence of templated insertion events in these cells.

We then analysed RI clones derived from p4.5 HPRT-2A-EGFP-2A-Puro-transfected *POLQ*-knockout cells. These Pol θ-independent junctions showed a totally different pattern from Lig4-independent junctions, with no apparent templated insertions and 42% of junctions (10 out of 24) showing 0–1 bp homology (Fig. 3a,b and Supplementary Data 2). Our data clearly suggest that 0–1 bp (or perhaps 0–2 bp) homology is characteristic of NHEJ-mediated RI, whereas templated insertion and 2–6 bp microhomology are Pol θ-dependent RI. These observations led us to revisit the junctions of RI events in wild-type cells. Notably, out of 60 junctions, 22 (37%) displayed templated insertions (≥6 bp repeats) and 6 (10%) relied on 2–6 bp microhomology (Fig. 3a,b and Supplementary Data 3), revealing that Pol θ-mediated end-joining does operate to cause RI even when NHEJ is functionally normal. It is interesting that whereas templated insertions appear favoured in wild-type cells, utilization of microhomology is prominent when Lig4 is absent (Fig. 3a). We also note that in RI events that present templated insertions, the location of template sequence is not just on a genome or vector sequence present nearby but also on a sequence around the opposite junction of the integration site in both wild-type and *LIG4*-knockout cells (Fig. 3c). These findings again highlight the intrinsic complexity of RI reactions, yet it is now proven that this complexity is totally attributed to Pol θ-mediated, but not NHEJ-mediated RI.

### Loss of Pol θ and Lig4 reveals homology-mediated RI

**Figure 4:**
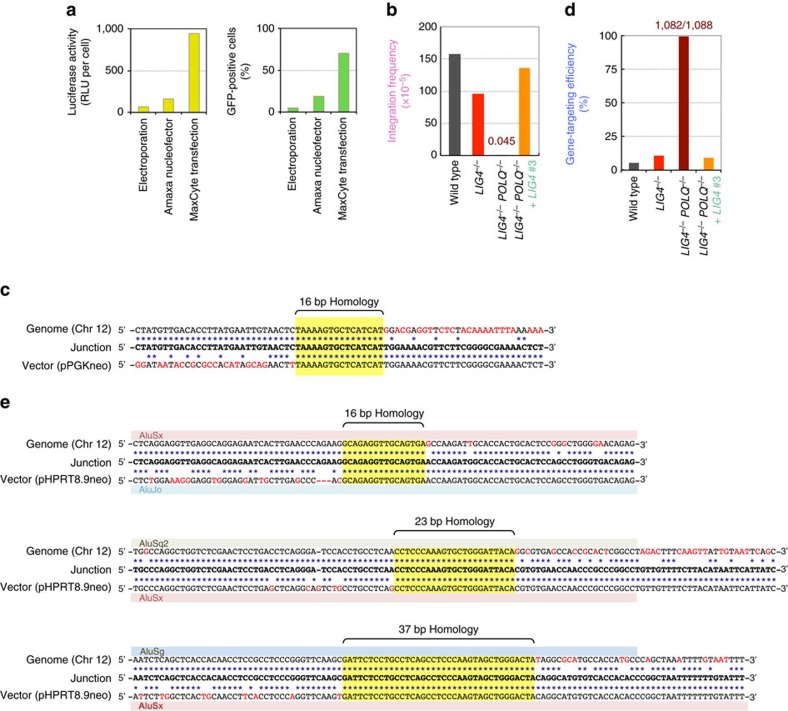
Increased transfection efficiency uncovers homology-mediated RI events. (**a**) Comparison of transfection efficiency between the three transfection methods. Transient expression assays were performed using a luciferase (left) or GFP (right) expression vector (see Supplementary Fig. 2l,m). (**b**) Integration frequency of MaxCyte transfected pPGKneo in wild-type, *LIG4*^*−/−*^, *LIG4*^*−/−*^*POLQ*^*−/−*^ and *LIG4*-complemented *LIG4*^*−/−*^*POLQ*^*−/−*^ cells. (**c**) Junction sequence in a rare RI clone obtained from *LIG4*^*−/−*^*POLQ*^*−/−*^ cells. (**d**) Gene-targeting efficiency, targeted integration frequency, and RI frequency of MaxCyte transfected pHPRT8.9-Neo2(−) in wild-type, *LIG4*^*−/−*^, *LIG4*^*−/−*^*POLQ*^*−/−*^ and *LIG4*-complemented *LIG4*^*−/−*^*POLQ*^*−/−*^ (#3) cells. (**e**) Junction sequences in rare RI clones obtained from *LIG4*^*−/−*^*POLQ*^*−/−*^ cells.

As we wished to further examine whether the dual loss of Pol θ and Lig4 completely abolish RI, we then employed a MaxCyte STX electroporation instrument for transfection. Transient expression assays showed that the MaxCyte system allows higher transfection efficiencies than other methods (Fig. 4a). Consistent with this expectation, RI frequencies in wild-type and *LIG4*-knockout cells were >10 times higher than those values shown above (Fig. 4b). When pPGKneo was MaxCyte transfected into double-knockout cells (10^8^ cells), five drug (G418)-resistant colonies appeared, meaning that the RI frequency was 4.5 × 10^−7^, not zero (Fig. 4b). These clones showed severe growth retardation, perhaps reflecting the occurrence of some sort of chromosomal rearrangements associated with vector integration; nevertheless, we were able to determine one junction, which intriguingly utilized a 16 bp region of homology between the vector (*Amp*^*r*^ in the vector backbone) and the genome (chromosome 12) (Fig. 4c), suggesting an SSA-type recombination. We next performed a gene-targeting assay using the promoter-containing *HPRT* targeting vector and found that the targeting efficiency in double-knockout cells was 99.4% (1,082/1,088), not 100% (Fig. 4d). Among the six RI clones, seven junctions analysed were all found to utilize homology-directed SSA-type recombination between two *Alu* elements, one of which resides in the homology arm of the vector and the other in the genome (chromosome 2 or 12) (Fig. 4e). Remarkably, these SSA-mediated RI events rely on ≥16 bp homology, a tendency clearly distinguished from Pol θ-mediated A-EJ that favours ≤6 bp microhomology. From these results, we conclude that even though non-homologous DNA integration is completely suppressed when Pol θ and Lig4 are both missing, SSA (or some other homology-mediated recombination) is capable of bringing about a very low level of RI under certain conditions.

### Role of Pol θ in chromosomal DSB repair

**Figure 5:**
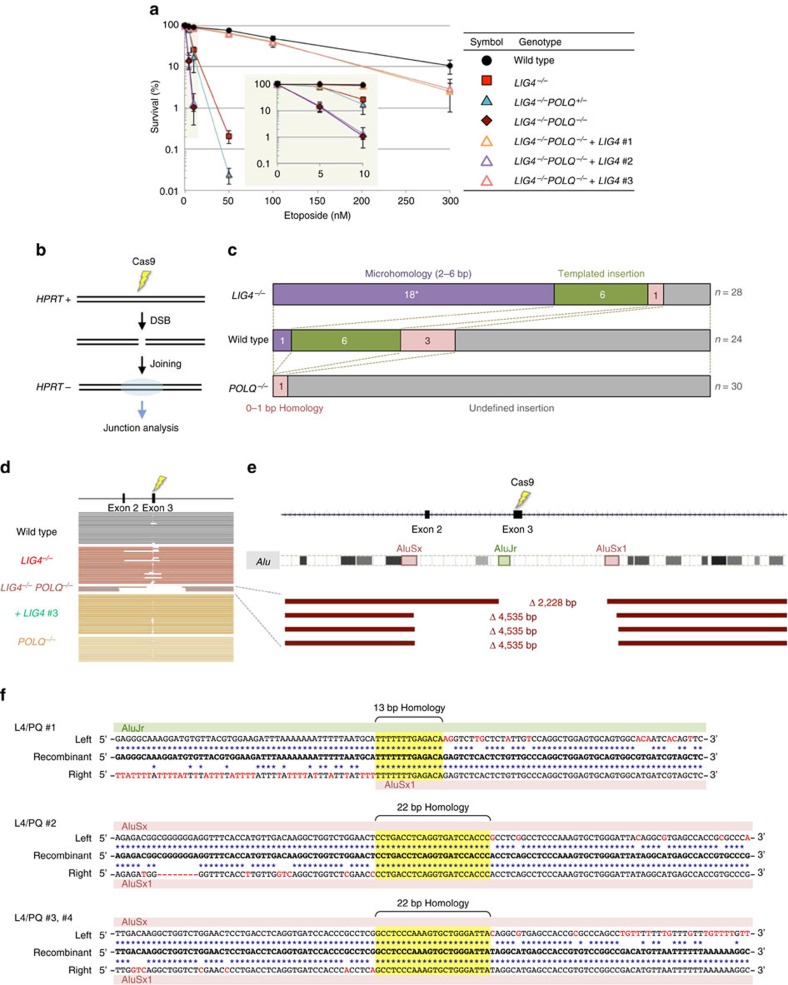
Impact of loss of Pol θ and Lig4 on chromosomal DSB repair. (**a**) Cellular sensitivity to etoposide. A clonogenic survival assay was performed with different concentrations of etoposide, a topoisomerase II inhibitor that induces chromosomal DSBs (mean±s.d.; *n*=3). Inset graph: Enlargement at low concentrations of etoposide. (**b**) Scheme for a Cas9-induced DSB joining assay. After DSB induction at the *HPRT* locus (exon 3), 6-thioguanine-resistant colonies (that is, *HPRT-*negative cells) were subjected to junction analysis. (**c**) Features and distribution of junctions obtained from wild-type, *LIG4*^*−/−*^ and *LIG4*-complemented *LIG4*^*−/−*^*POLQ*^*−/−*^ (#3) cells. The features were classified into four categories; microhomology (2–6 bp homology), templated insertion (≥6 bp direct or inverted repeats), undefined insertion (1–12 bp) and 0–1 bp homology. A Fisher’s exact test was used to determine statistical significance. (*P<0.00001). Although not indicated, the absence of templated insertion in *POLQ*^*−/−*^ cells is statistically significant (*P*=0.0052). (**d**) Schematic representation of recombinants obtained from wild-type, *LIG4*^*−/−*^, *LIG4*^*−/−*^*POLQ*^*−/−*^, *LIG4*-complemented *LIG4*^*−/−*^*POLQ*^*−/−*^ (#3) and *POLQ*^*−/−*^ cells. (**e**) Schematic representation of four recombinants obtained from *LIG4*^*−/−*^*POLQ*^*−/−*^ cells. The locations of *Alu* elements at the *HPRT* locus are also indicated. (**f**) Junction sequences in the four recombinants obtained from *LIG4*^*−/−*^*POLQ*^*−/−*^ cells.

Finally, we examined the importance of human Pol θ in chromosomal DSB repair. As shown in Fig. 5a, loss of Pol θ caused only a small or no increase in cellular sensitivity to etoposide (a topoisomerase II inhibitor that induces DSBs^41^) in wild-type cells, but significantly augmented etoposide hypersensitivity of *LIG4*-knockout cells. These results indicate that Pol θ only plays a minor role in repair of DSBs and suggest that the absence of both A-EJ and NHEJ leads to severe compromise in non-homologous DSB repair. To further confirm this, we employed CRISPR-Cas9 technology^42^ to induce a DSB at the *HPRT* locus and look at rejoining of the chromosomal DSB (Fig. 5b). As summarized in Fig. 5c and Supplementary Data 4, neither microhomology nor templated insertion was observed in cells lacking Pol θ (*n*=49; with only one exception having 2 bp microhomology, which is reasonably attributable to NHEJ repair^11^), clearly showing that Pol θ is indeed indispensable for A-EJ. In sharp contrast, *LIG4*-independent junctions were associated with larger terminal deletions (Fig. 5d) and 24 out of 28 (86%) relied on microhomology (2–6 bp homology) or templated insertion (≥;6 bp repeats) (Fig. 5c and Supplementary Data 4). These results show that Pol θ (and hence A-EJ) efficiently comes to the fore when NHEJ cannot deal with the DSB. In double-knockout cells, the efficiency of DSB joining was significantly reduced (Supplementary Data 4) and all junctions analysed accompanied large (>2 kb) terminal deletions (Fig. 5d,e), a finding consistent with earlier work in *Drosophila*^43^. Notably, these junctions all displayed clear traits of SSA-type recombination between two separate *Alu* elements (Fig. 5f), similar to that seen in rare RI clones described above.

## Discussion

Pol θ is a unique DNA polymerase in that it is highly expressed in various types of cancer^44^,^45^,^46^. Intriguingly, loss of Pol θ is synthetically lethal with HR defects^33^,^47^ and thus developing Pol θ inhibitors will lead to a new therapeutic agent for various cancers with compromised HR^40^,^48^. Most remarkably, in this study, we have demonstrated ‘synthetic abolishment of RI’ by the dual loss of Pol θ and Lig4 (Supplementary Fig. 4a). Tijsterman and coworkers^49^ have very recently reached the same conclusion using mouse embryonic stem cells deficient in Pol θ and NHEJ. We emphasize that complete or even near-complete suppression of RI in mammalian cells is highly intriguing in light of the intrinsic complexity of RI as well as the long history of gene targeting struggling with unwanted RI clones, even despite recent advances in artificial nucleases, which stimulate RI as well as targeted integration of a donor DNA^50^. We propose that simultaneous inhibition of Pol θ and NHEJ will provide a novel strategy for ultra-efficient gene targeting even without using artificial nucleases.

Mechanistically, we have presented evidence that A-EJ depends crucially on Pol θ and this form of DSB repair is typically characterized by 2–6 bp microhomology or templated insertions at the junctions. A similar preference was recently observed in Pol θ-mediated end-joining in several mammalian cell types^51^,^52^, consistent with a unique property of Pol θ to stabilize the annealing of two single-stranded DNA molecules with as little as 2 bp of homology^31^,^53^. Intriguingly, the relative usage of microhomology versus templated insertion was similar between RI and chromosomal DSB repair when Lig4 is absent (Figs 1b and 5c). Clearly, however, the importance of Pol θ in these processes appears to be distinct: a minor role in DSB repair and a substantial contribution to RI even in the presence of NHEJ (Supplementary Fig. 4b). This implies that there exists an unknown mechanism that prevents NHEJ from causing RI, allowing a larger contribution of Pol θ. This notion is well supported by our observations that A-EJ usage was marginal in chromosomal DSB repair, whereas templated insertions were frequently observed in RI clones from wild-type cells. Finally, it should also be emphasized that we have shown for the first time that the dual loss of Lig4 and Pol θ reveals SSA-type recombination that was not seen when either Lig4 or Pol θ was present. As Pol θ facilitates the formation of an annealed intermediate using as little as 2 bp of homology^31^,^53^, more extensive resection of DNA ends would be prevented, thereby minimizing the occurrence of deleterious DSB repair by SSA. Mechanistic differences of A-EJ and SSA have long been elusive, particularly in terms of homology requirements^22^. In this study, we have shown in both RI assay and chromosomal DSB joining assay that SSA-type recombination relies on ≥13 bp homology, which contrasts with the 2–6 bp preference seen in A-EJ. Our findings described here shed light on the mechanism of SSA (between *Alu* elements), which, albeit normally suppressed, has been reported to cause deleterious outcomes involving deletions and translocations^54^,^55^,^56^.

## Methods

### Plasmids

Targeting vectors were constructed using the MultiSite Gateway system (Life Technologies) to assemble two homology arms and a drug-resistance gene cassette^35^,^37^,^57^. To generate targeting vectors for the human *POLQ* gene, 3.0 and 5.3 kb *POLQ* genomic fragments were PCR amplified with Tks Gflex DNA Polymerase (Takara Bio) using Nalm-6 genomic DNA as a template and were used as 5′- and 3′-arms, respectively. The primers used were: POLQ 5′ Fw (5′-GGGGACAACTTTGTATAGAAAAGTTGGGCTCAGTACCATGTTACAGAGG-3′) and POLQ 5′ Rv (5′-GGGGACTGCTTTTTTGTACAAACTTGTAGTAGTTCCATGTTGTGCCAGC-3′) for the 5′-arm and POLQ 3′ Fw (5′-GGGGACAGCTTTCTTGTACAAAGTGGGTGCACTACTGTGGCAGACCTTGCTAGAGC-3′) and POLQ 3′ Rv (5′-GGGGACAACTTTGTATAATAAAGTTGCTATATTACCCTGTTATCCCTAGCGTAACTCATTGGAACAAGTCAACTGCTGG-3′) for the 3′-arm. A floxed hygromycin- or puromycin-resistance gene was placed between 5′- and 3′-arms, thus yielding targeting vectors pPOLQ 2A-Hyg and pPOLQ 2A-Puro. An *AAVS1* targeting vector, pAAVS1 Neo, was constructed using the MultiSite Gateway system to assemble two homology arms (4.4 and 3.1 kb) and a neomycin-resistance gene cassette. Genomic fragments for homology arms were PCR amplified with *att*B-containing primers (AAVS1 5′ Fw (5′-GGGGACAACTTTGTATAGAAAAGTTGGCTTTGCCACCCTATGCTGACACC-3′) and AAVS1 5′ Rv (5′-GGGGACTGCTTTTTTGTACAAACTTGCAAGAAGCGCACCACCTCCAGGTTC-3′) for the 5′-arm and HPRT 3′ Fw (5′-GGGGACAGCTTTCTTGTACAAAGTGGAACATCGCCGCCGTCAACAGTGAC-3′) and HPRT 3′ Rv (5′-GGGGACAACTTTGTATAATAAAGTTGCTATATTACCCTGTTATCCCTAGCGTAACTCAGAGGACTTTGCGGTGTTGGAGG-3′) for the 3′-arm). A *PRKDC* targeting vector, pPK-Hyg, was designed to replace exons 8–10 with a floxed hygromycin-resistance gene. Briefly, 2.1 and 5.1 kb *PRKDC* genomic fragments were PCR amplified with *att*B-containing primers (PK 5′ Fw (5′-GGGGACAACTTTGTATAGAAAAGTTGAGAGGTGGAGCACACGACCCACGGG-3′) and PK 5′ Rv (5′-GGGGACTGCTTTTTTGTACAAACTTGCAGTCCACTCTCCTGCAGCCCCTCC -3′) for the 5′-arm and PK 3′ Fw (5′-GGGGACAGCTTTCTTGTACAAAGTGGTGGTCGGCTGAGGAGACCGTGCTTG -3′) and PK 3′ Rv (5′-GGGGACAACTTTGTATAATAAAGTTGCGAGGATCTGATGGTGCACCTCTTC -3′) for the 3′-arm). A *RAD54B* targeting vector, p7.8 RAD54B Hyg, was designed to replace exons 5 and 6 with a floxed hygromycin-resistance gene. Briefly, 3.3 and 4.5 kb *RAD54B* genomic fragments were PCR amplified with *att*B-containing primers (R54B 5′ Fw (5′-GGGGACAACTTTGTATAGAAAAGTTGTTAATTAATGGTTTATGCTAGCTCAGTGAAGC-3′) and R54B 5′ Rv (5′-GGGGACAGCTTTCTTGTACAAAGTGGGTGGTTCTTGATAGATGCCACGTG-3′) for the 5′-arm and R54B 3′ Fw (5′-GGGGACAGCTTTCTTGTACAAAGTGGATGGACCCTACTGGAAGTCAGTTGG-3′) and R54B 3′ Rv (5′-GGGGACAACTTTGTATAATAAAGTTGCTATATTACCCTGTTATCCCTAGCGTAACTAGGCTAATGAGGGCTGTAGTTGTC-3′) for the 3′-arm). A *RAD54* targeting vector, p6.5 RAD54 2A-Puro, was designed to replace exons 4–7 with a floxed puromycin-resistance gene. Briefly, 2.9 and 3.6 kb *RAD54* genomic fragments were PCR amplified with *att*B-containing primers (R54 5′ Fw (5′-GGGGACAACTTTGTATAGAAAAGTTGGAATTCGGGGACTTTGAAAGGCTTTGAC-3′) and R54 5′ Rv (5′-GGGGACAGCTTTCTTGTACAAAGTGGGTGGTTCTTGATAGATGCCACGTG-3′) for the 5′-arm and R54 3′ Fw (5′-GGGGACAGCTTTCTTGTACAAAGTGGGCCAGAGTCCAGAGTGCAAGCCAG-3′) and R54 3′ Rv (5′-GGGGACAACTTTGTATAATAAAGTTGCTATATTACCCTGTTATCCCTAGCGTAACTAAAAGCGTTACTGGGAGGAAGATG-3′) for the 3′-arm). A *TP53BP1* targeting vector, p2.4 53BP1 IRES-Hyg, was designed to replace exon 2 with a floxed hygromycin-resistance gene. Briefly, 0.9 and 1.5 kb *TP53BP1* genomic fragments were PCR amplified with *att*B-containing primers (53BP1 5′ Fw (5′-GGGGACAACTTTGTATAGAAAAGTTGGCAGCTGTTCTCTTTACCATGGAG-3′) and 53BP1 5′ Rv (5′-GGGGACTGCTTTTTTGTACAAACTTGTTCCAGTAGGGTCCATCTGCTCC-3′) for the 5′-arm and 53BP1 3′ Fw (5′-GGGGACAGCTTTCTTGTACAAAGTGGATGGACCCTACTGGAAGTCAGTTGG-3′) and 53BP1 3′ Rv (5′-GGGGACAACTTTGTATAATAAAGTTGCTATATTACCCTGTTATCCCTAGCGTAACTCAAGTCCCATTTCCTAATCCAC-3′) for the 3′-arm). An *HPRT* targeting vector, pHPRT8.9-Neo2(−), was constructed by inserting a neomycin-resistance gene cassette into the XhoI site of an 8.9 kb *HPRT* fragment^58^. The entire DNA sequence of pHPRT8.9-Neo2(−) has been deposited in the EMBL/DDBJ/GenBank database under accession number LC269955. The sequence of the p4.5 HPRT-2A-EGFP-2A-Puro vector^35^ is also deposited in the database under accession number LC270125. All the targeting vectors were purified with QIAGEN Plasmid *Plus* Midi Kits (QIAGEN K.K.) and linearized with appropriate restriction enzyme before transfection^57^.

### Cell culture

Nalm-6 cells were cultured in a 5% CO_2_ incubator at 37 °C in MEM medium (Nissui Seiyaku) supplemented with 10% calf serum (Hyclone), 2 mM MEM non-essential amino acids (Wako Pure Chemical), 1 mM sodium pyruvate (Wako Pure Chemical), 50 μM 2-mercaptoethanol (Wako Pure Chemical) and 0.15 μM Vitamin B12 (Sigma-Aldrich). Nalm-6 is a human pre-B cell line available from ATCC and was kindly provided by Dr Michael R. Lieber. The Nalm-6 cell line was originally established from the peripheral blood of a 19-year-old man with acute lymphoblastic leukaemia^59^ and has a stable near-diploid karyotype^60^. All cell lines used in this study are not listed in the database of commonly misidentified cell lines maintained by ICLAC and were tested for mycoplasma contamination using an e-Myco Mycoplasma PCR Detection Kit (iNtRON Biotechnology, Inc.).

### Transfection

DNA transfection using the GTE-1 electroporation apparatus (Shimadzu) was performed according to the manufacturer’s instructions. Briefly, cells were washed twice with Saline G (130 mM NaCl, 5.3 mM KCl, 1.1 mM Na_2_HPO_4_, 1.1 mM KH_2_PO_4_, 6.1 mM glucose, 0.49 mM MgCl_2_ and 0.9 mM CaCl_2_) and an aliquot of the cell suspension (4 × 10^6^ cells in 40 μl of Saline G) was electroporated with 4 μg of plasmid DNA. The cells were then cultured for 24 h and replated at a density of 0.5–1 × 10^6^ cells per 90 mm dish into agarose medium containing hygromycin B (0.3–0.5 mg ml^−1^, Wako Pure Chemical), puromycin (0.15–0.5 μg ml^−1^, Wako Pure Chemical) or G418 sulfate (1.0 mg ml^−1^, Wako Pure Chemical). Meanwhile, small aliquots of the transfected cells were replated into drug-free agarose medium to determine the plating efficiency. DNA transfection (Nucleofection) using the Nucleofector II system (Lonza) was performed according to the manufacturer’s instructions. Briefly, 2 × 10^6^ cells were suspended with 100 μl of the supplied solutions (Solution T) and transfected with 2 μg of linearized vector. DNA transfection using the MaxCyte STX device (MaxCyte) was performed according to the manufacturer’s instructions. Briefly, 5 × 10^7^ cells were suspended with 400 μl of the supplied solutions (MaxCyte Electroporation Buffer) and transfected with 50 μg of linearized vector. Following transfection, cells were incubated at 37 °C and 5% CO_2_ for 20 min to recover. Cells were then resuspended to ∼5 × 10^5^ cells per ml in culture medium and incubated for 24 h.

### Generation of human *POLQ*-knockout cell lines

The human *POLQ* targeting vector pPOLQ 2A-Hyg was transfected into wild-type and *LIG4*^*−/−*^ Nalm-6 cells^37^ and hygromycin-resistant colonies were isolated and expanded to prepare genomic DNA. Gene-targeting events were screened by PCR analysis using primers POLQ 5′ ext (5′-CTGAGAGACAGCATCGACAAATGG-3′) and Universal primer 2A (5′-CACCGCATGTTAGAAGACTTCCTC-3′). Subsequently, pPOLQ 2A-Puro was transfected into *POLQ*^*+/−*^ and *LIG4*^*−/−*^*POLQ*^*+/−*^ cells, and puromycin-resistant clones were subjected to PCR analysis using primers POLQ-N Fw (5′-GTGCAGCTATTGAGCTCAGCAACC-3′) and POLQ-N Rv (5′-GATGCCAAACGTAAGACGCTTCTG-3′). The disruption of the *POLQ* gene was further confirmed by PCR analysis using primers POLQ 5′ext and POLQ exon 17 Rv (5′-ACCACCAAGGTGTCATCACAACC-3′), and by RT–PCR analysis using primers POLQ 5′ext and POLQ-N Rv for *POLQ*, and GAPDH RT Fw (5′-GCTGGCGCTGAGTACGTCGTGGAG-3′) and GAPDH RT Rv (5′-CTTACTCCTTGGAGGCCATGTGGG-3′) for *GAPDH*. RNA extraction was performed using TRIzol reagent (Life Technologies) according to the manufacturer’s instructions. To restore *LIG4* expression in *LIG4*^*−/−*^*POLQ*^*−/−*^ cells, an EF1α promoter-driven *LIG4* expression vector^61^ (kindly provided by Dr Yoshihisa Matsumoto) was linearized with ScaI and transfected into *LIG4*^*−/−*^*POLQ*^*−/−*^ cells, which were then selected with 0.4 μg ml^−1^ puromycin. After a 2-week cultivation, the resulting colonies were picked and subjected to western blot analysis.

### Western blot analysis

Cells were rinsed twice with PBS^−^ and scraped in 200 μl of lysis buffer (50 mM Tris-HCl (pH 6.8), 2% sodium dodecylsulfate, 10% glycerol, 100 μM dithiothreitol, 1 mM phenylmethylsulfonyl fluoride) containing Protease Inhibitor Cocktail (1/10 volume of buffer; Sigma-Aldrich). The lysates were allowed to stand for 20 min at 4 °C and, after sonication, centrifuged for 30 min at 16,000 *g*. The supernatants were collected and used for western blot analysis. Twenty micrograms of the lysates were electrophoresed in a 7.5% polyacrylamide gel and then transferred onto a polyvinylidene difluoride membrane (Millipore). The membrane was probed with anti-Lig4 antibody (1:500, kindly provided by Dr Hirobumi Teraoka)^37^ or anti-Actin antibody (1:2,000, A2066, Sigma-Aldrich). Signals were detected with Clarity Western ECL Substrate (Bio-Rad) and analysed using a Fuji Image Analyzer LAS-1000UVmini (Fuji Film Co.). Full scans of the blots are shown in Supplementary Fig. 2.

### Luciferase assay

To construct a NanoLuc Luciferase (Nluc) expression vector, the open reading frame of a gene encoding *Nluc* was excised from pNL1.1[Nluc] vector (Promega) and a 0.9 kb BamHI/NheI fragment containing *Nluc* gene was inserted into BamHI/NheI-digested pIRES (Clontech), thus yielding pCMV-Nluc. Transfection of the pCMV-Nluc vector was performed using GTE-1 electroporation, Nucleofection, or MaxCyte transfection. After a 24 h incubation, an aliquot (5 × 10^4^ cells) was suspended in 50 μl of growth medium and luciferase activity was measured using the Nano-Glo Luciferase Assay System (Promega) according to the manufacturer’s instructions.

### Flow cytometric analysis

Cells were transfected with pmaxGFP (Lonza) by either GTE-1 electroporation, Nucleofection or MaxCyte transfection. After a 24 h incubation, green fluorescent protein (GFP)-positive cells were enumerated using a JSAN cell sorter (Bay Bioscience).

### RI and gene-targeting assays

For RI assays, pPGKneo^62^ was linearized and transfected into Nalm-6 cells. After cultivation for 2–3 weeks, the resulting G418-resistant colonies were counted and the total integration frequency was calculated by dividing the number of G418-resistant colonies with that of surviving cells. For gene-targeting assays, each targeting vector was linearized and transfected into Nalm-6 cells. After a 2–3 week incubation, genomic DNA was isolated from drug-resistant colonies and subjected to PCR analysis. The gene-targeting efficiency was calculated by dividing the number of targeted clones with that of drug-resistant clones analysed. The targeted integration frequency was calculated by multiplying the total integration frequency by the targeting efficiency. The RI frequency was calculated by subtracting the targeted integration frequency from the total integration frequency.

### Junction analysis of RI clones

The p4.5 HPRT-2A-EGFP-2A-Puro targeting vector was transfected into Nalm-6 wild-type, *LIG4*^*−/−*^ or *POLQ*^*−/−*^ cells and the resulting RI clones (50 clones from wild type, 25 clones from *LIG4*^*−/−*^ and 16 clones from *POLQ*^*−/−*^ cells) were subjected to junction analysis based on inverse PCR. Briefly, 10 μg of genomic DNA extracted from RI clones was digested with 30 U of HindIII (for 5′-junction analysis) or BamHI and BclI (for 3′-junction analysis). The digested DNA was precipitated with ethanol and 1 μg of the DNA was self-circularized in 400 μl of ligation buffer at 16 °C overnight by using T4 DNA Ligase (Takara Bio). The self-ligated DNA was extracted with phenol:chloroform and then with chloroform alone. Subsequently, the self-ligated DNA was precipitated with ethanol and used as a template for PCR amplification. PCR reactions were performed with Tks Gflex DNA Polymerase (Takara Bio) or EmeraldAmp PCR Master Mix (Takara Bio). Primers used to amplify 5′-junctions were as follows: iPCR-EGFP Rv, 5′-TGAACTTGTGGCCGTTTACGTCGC-3′; iPCR-5-HPRT1 Fw, 5′-TCACATCTCGAGCAAGACGTTCAG-3′; iPCR-5-HPRT2 Fw, 5′-CAGAGTCCCACTATACCACAATG-3′; iPCR-5-HPRT3 Fw, 5′-CACCTGACGTCTAAGAAACC-3′. Primers used to amplify 3′-junctions were as follows: iPCR-EGFP Fw, 5′-AACGAGAAGCGCGATCACATGGTC-3′; iPCR-3-HPRT1 Rv, 5′-CAGACTGAAGAGCTATTGTG-3′; iPCR-3-HPRT2 Rv, 5′-GCATCACAACATTGACACTGTGGATG-3′; iPCR-3-HPRT3 Rv, 5′-GGGCTATGCAAGGAAGATATACTG-3′. The PCR products were cloned into the pTAKN2 T-Vector (BioDynamics Laboratory Inc.) to determine the sequence (Eurofins Genomics K.K.). BLAST programmes were used to map the junction sequences onto the human genome (University of California Santa Cruz’s Genome Browser). Sequence alignments were performed using a computer software GENETYX-MAC (Software Development Co.). Sequence features (microhomologies and template insertions involving direct or inverted repeats) were identified by visual inspection. It is noteworthy that we only regarded ≥6 bp direct or inverted repeats as traits of templated insertion and hence <6 bp repeats were not taken into account as those can be frequently seen around the junctions and we could not fully rule out the possibility that <6 bp repeats at the junctions were not associated with templated insertion. Thus, the numbers of templated insertion could be even underestimated in our analysis. Furthermore, those clones that were picked independently but had identical sequences at each junction were regarded as the same recombinant cell line. Likewise, those clones in which only one side of the junctions was determined (including those showing vector-vector joining (concatemerization)) were not taken into account in the analysis, because it was unclear whether or not the inserted nucleotides relied on templated insertions using the other side of the junctions.

### Drug sensitivity assay

To examine cellular sensitivity to etoposide, clonogenic survival assays were performed by plating exponentially growing cells, at a density of 10^2^–10^6^ cells per 60 mm dish, into agarose medium containing various concentrations of etoposide (BioVision, Inc.). After a 2–3 week incubation at 37 °C, visible colonies were counted and the per cent survival was determined by comparing the number of surviving colonies to untreated controls.

### Chromosomal DSB joining assay

An all-in-one CRISPR vector, pX330-U6-Chimeric_BB-CBh-hSpCas9 (pX330, Addgene plasmid #42230), was used to express Cas9 and single-guide RNA. Sense and antisense oligonucleotides of each single-guide RNA targeting sequence were annealed and cloned into pX330 at the BbsI site. The CRISPR-Cas9 target sequences (20 bp target and 3 bp PAM sequence (underlined)) used in this study include: pHPRT CRISPR1, 5′-GATGTGATGAAGGAGATGGGAGG-3′; pHPRT CRISPR2, 5′-AGTCCTACAGAAATAAAATCAGG-3′. After transfection, cells were cultured for 1 week and then selected with 20 μM 6-thioguanine. The resulting drug-resistant (*HPRT*-negative) colonies were isolated and expanded to prepare genomic DNA. To exclude spontaneous mutants irrelevant to Cas9 cleavage, PCR analysis was performed using two sets of primers HPRT-F (5′-TGAGGGCAAAGGATGTGTTACGTG-3′) and HPRT US Rv (5′-CAGTCCTACAGCCCTAAAATCAGG-3′), and HPRT DS Fw (5′-GAGATGTGATGAAGGAGATGGGAG-3′) and HPRT 0.7 Rv (5′-TCCACAGTGTCAATGTTGTGATGC-3′). Subsequently, flanking sequences were PCR amplified with primers HPRT-F and HPRT 0.7 Rv or HPRT 2.0 Fw (5′-CAGCAGCTGTTCTGAGTACTTGCT-3′) and HPRT 2.3 Rv (5′-ACAGTATATCTTCCTTGCATAGCC-3′) or HPRT 3.8 Fw (5′-CACATCACAGGTACCATATCAGTG-3′) and HPRT 2.8 Rv (5′-CAGGGTAGAAATGCTACTTCAGGC-3′). PCR reactions were performed with ExTaq DNA polymerase (Takara Bio) or EmeraldAmp PCR Master Mix (Takara Bio) and PCR products were subcloned into the pGEM-T Easy vector (Promega) to determine the sequence (Eurofins Genomics K.K). Sequence alignments were performed using a computer software GENETYX-MAC (Software Development Co.). Sequence features (microhomologies and template insertions involving direct or inverted repeats) were identified by visual inspection and defined as above (*cf*. junction analysis of RI clones). During data analysis, the pCRISPR HPRT2 plasmid was found to be significantly less efficient in cleaving DNA than pCRISPR HPRT1. For this reason, those recombinants that retained the pCRISPR HPRT1 target site were excluded from the data sets. Furthermore, those clones that were picked independently but had identical junction sequences (for example, recombinants #3 and #4 from *LIG4*^*−/−*^*POLQ*^*−/−*^ cells) were regarded as independent clones and thus all the recombinants analysed are included in the data sets shown in Fig. 5 and Supplementary Data 4.

### Data availability

The data supporting the findings of this study are available within the article and its Supplementary Information, or from the authors upon reasonable request.

## Additional information

**How to cite this article:** Saito, S. *et al*. Dual loss of human *POLQ* and *LIG4* abolishes random integration. *Nat. Commun.* **8,** 16112 doi: 10.1038/ncomms16112 (2017).

**Publisher’s note:** Springer Nature remains neutral with regard to jurisdictional claims in published maps and institutional affiliations.

## Supplementary Material

Supplementary Information

Supplementary Data 1

Supplementary Data 2

Supplementary Data 3

Supplementary Data 4

Peer review file

## Figures and Tables

**Table 1:** Summary of gene-targeting assays in *LIG4* ^*−/−*^ *POLQ* ^*−/−*^ cells.

**Target locus**	**Targeting vector**			**No. of cells treated ( × 10**^**6**^**)**	**Gene-targeting efficiency**
	**5′-arm**	**3′-arm**	Selection marker*		
*HPRT*	3.8 kb	5.1 kb	P_*PGK*_ -Neo	40.0	100% (138/138)
*HPRT*	1.7 kb	2.8 kb	2A-Puro	24.0	100% (59/59)
*AAVS1*	4.4 kb	3.1 kb	P_*PGK*_ -Neo	4.0	100% (8/8)
*PRKDC*	2.1 kb	5.1 kb	P_*PGK*_ -Hyg	8.0	100% (3/3)
*RAD54B*	3.3 kb	4.5 kb	P_*PGK*_ -Hyg	8.0	100% (5/5)
*RAD54L*	2.9 kb	3.6 kb	2A-Puro	8.0	100% (1/1)
*TP53BP1*	0.9 kb	1.5 kb	IRES-Hyg	8.0	100% (2/2)

P_*PGK*_, PGK promoter; 2A, 2A peptide sequence.

^*^It is noteworthy that although *Hyg*^*r*^ and *Puro*^*r*^ (hygromycin- and puromycin-resistance gene, respectively) have already been used to disrupt *POLQ*, increasing drug concentrations allowed us to reuse these genes as gene-targeting selection markers.

## References

[b1] Smithies O., Koralewski M. A., Song K. Y. & Kucherlapati R. S. Homologous recombination with DNA introduced into mammalian cells. Cold Spring Harb. Symp. Quant. Biol. 49, 161–170 (1984). 6597754 10.1101/sqb.1984.049.01.019

[b2] Capecchi M. R. Altering the genome by homologous recombination. Science 244, 1288–1292 (1989). 2660260 10.1126/science.2660260

[b3] Roth D. B. & Wilson J. H. Relative rates of homologous and nonhomologous recombination in transfected DNA. Proc. Natl Acad. Sci. USA 82, 3355–3359 (1985). 2987922 10.1073/pnas.82.10.3355 PMC397774

[b4] Ninomiya Y., Suzuki K., Ishii C. & Inoue H. Highly efficient gene replacements in Neurospora strains deficient for nonhomologous end-joining. Proc. Natl Acad. Sci. USA 101, 12248–12253 (2004). 15299145 10.1073/pnas.0402780101 PMC514464

[b5] Lieber M. R. The mechanism of double-strand DNA break repair by the nonhomologous DNA end-joining pathway. Annu. Rev. Biochem. 79, 181–211 (2010). 20192759 10.1146/annurev.biochem.052308.093131 PMC3079308

[b6] Sado K. . Identification of a mutated DNA ligase IV gene in the X-ray-hypersensitive mutant SX10 of mouse FM3A cells. J. Biol. Chem. 276, 9742–9748 (2001). 11133995 10.1074/jbc.M010530200

[b7] Iiizumi S. . Impact of non-homologous end-joining deficiency on random and targeted DNA integration: implications for gene targeting. Nucleic Acids Res. 36, 6333–6342 (2008). 18835848 10.1093/nar/gkn649 PMC2577324

[b8] Ishii A., Kurosawa A., Saito S. & Adachi N. Analysis of the role of homology arms in gene-targeting vectors in human cells. PLoS ONE 9, e108236 (2014). 25250686 10.1371/journal.pone.0108236 PMC4176728

[b9] Takata M. . Homologous recombination and non-homologous end-joining pathways of DNA double-strand break repair have overlapping roles in the maintenance of chromosomal integrity in vertebrate cells. EMBO J. 17, 5497–5508 (1998). 9736627 10.1093/emboj/17.18.5497 PMC1170875

[b10] Kurosawa A. . DNA ligase IV and Artemis act cooperatively to suppress homologous recombination in human cells: implications for DNA double-strand break repair. PLoS ONE 8, e72253 (2013). 23967291 10.1371/journal.pone.0072253 PMC3743779

[b11] Pannunzio N. R., Li S., Watanabe G. & Lieber M. R. Non-homologous end joining often uses microhomology: implications for alternative end joining. DNA Repair (Amst) 17, 74–80 (2014). 24613510 10.1016/j.dnarep.2014.02.006 PMC4440676

[b12] Williams G. J. . Structural insights into NHEJ: building up an integrated picture of the dynamic DSB repair super complex, one component and interaction at a time. DNA Repair (Amst) 17, 110–120 (2014). 24656613 10.1016/j.dnarep.2014.02.009 PMC4102006

[b13] Chang H. H. Y., Pannunzio N. R., Adachi N. & Lieber M. R. Non-homologous DNA end joining and alternative pathways to double-strand break repair. Nat. Rev. Mol. Cell Biol. doi:; DOI: 10.1038/nrm.2017.48 (2017). PMC7062608 28512351

[b14] Boboila C., Alt F. W. & Schwer B. Classical and alternative end-joining pathways for repair of lymphocyte-specific and general DNA double-strand breaks. Adv. Immunol. 116, 1–49 (2012). 23063072 10.1016/B978-0-12-394300-2.00001-6

[b15] Ceccaldi R., Rondinelli B. & D'Andrea A. D. Repair pathway choices and consequences at the double-strand break. Trends Cell Biol. 26, 52–64 (2016). 26437586 10.1016/j.tcb.2015.07.009 PMC4862604

[b16] Jasin M. & Rothstein R. Repair of strand breaks by homologous recombination. Cold Spring Harb. Perspect. Biol. 5, a012740 (2013). 24097900 10.1101/cshperspect.a012740 PMC3809576

[b17] Iliakis G., Murmann T. & Soni A. Alternative end-joining repair pathways are the ultimate backup for abrogated classical non-homologous end-joining and homologous recombination repair: implications for the formation of chromosome translocations. Mutat. Res. Genet. Toxicol. Environ. Mutagen 793, 166–175 (2015). 26520387 10.1016/j.mrgentox.2015.07.001

[b18] Sfeir A. & Symington L. S. Microhomology-mediated end joining: a back-up survival mechanism or dedicated pathway? Trends Biochem. Sci. 40, 701–714 (2015). 26439531 10.1016/j.tibs.2015.08.006 PMC4638128

[b19] Sartori A. A. . Human CtIP promotes DNA end resection. Nature 450, 509–514 (2007). 17965729 10.1038/nature06337 PMC2409435

[b20] Mimitou E. P. & Symington L. S. DNA end resection: many nucleases make light work. DNA Repair (Amst) 8, 983–995 (2009). 19473888 10.1016/j.dnarep.2009.04.017 PMC2760233

[b21] Truong L. N. . Microhomology-mediated end joining and homologous recombination share the initial end resection step to repair DNA double-strand breaks in mammalian cells. Proc. Natl Acad. Sci. USA 110, 7720–7725 (2013). 23610439 10.1073/pnas.1213431110 PMC3651503

[b22] Bhargava R., Onyango D. O. & Stark J. M. Regulation of single-strand annealing and its role in genome maintenance. Trends Genet. 32, 566–575 (2016). 27450436 10.1016/j.tig.2016.06.007 PMC4992407

[b23] Callinan P. A. & Batzer M. A. Retrotransposable elements and human disease. Genome Dyn. 1, 104–115 (2006). 18724056 10.1159/000092503

[b24] Treangen T. J. & Salzberg S. L. Repetitive DNA and next-generation sequencing: computational challenges and solutions. Nat. Rev. Genet. 13, 36–46 (2011). 22124482 10.1038/nrg3117 PMC3324860

[b25] Yu A. M. & McVey M. Synthesis-dependent microhomology-mediated end joining accounts for multiple types of repair junctions. Nucleic Acids Res. 38, 5706–5717 (2010). 20460465 10.1093/nar/gkq379 PMC2943611

[b26] Yousefzadeh M. J. . Mechanism of suppression of chromosomal instability by DNA polymerase POLQ. PLoS Genet. 10, e1004654 (2014). 25275444 10.1371/journal.pgen.1004654 PMC4183433

[b27] Cho N. W. & Greenberg R. A. DNA repair: familiar ends with alternative endings. Nature 518, 174–176 (2015). 25642961 10.1038/nature14200

[b28] Seki M. . High-efficiency bypass of DNA damage by human DNA polymerase Q. EMBO J. 23, 4484–4494 (2004). 15496986 10.1038/sj.emboj.7600424 PMC526458

[b29] Arana M. E., Seki M., Wood R. D., Rogozin I. B. & Kunkel T. A. Low-fidelity DNA synthesis by human DNA polymerase theta. Nucleic Acids Res. 36, 3847–3856 (2008). 18503084 10.1093/nar/gkn310 PMC2441791

[b30] Yousefzadeh M. J. & Wood R. D. DNA polymerase POLQ and cellular defense against DNA damage. DNA Repair (Amst) 12, 1–9 (2013). 23219161 10.1016/j.dnarep.2012.10.004 PMC3534860

[b31] Newman J. A., Cooper C. D., Aitkenhead H. & Gileadi O. Structure of the helicase domain of DNA polymerase theta reveals a possible role in the microhomology-mediated end-joining pathway. Structure 23, 2319–2330 (2015). 26636256 10.1016/j.str.2015.10.014 PMC4671958

[b32] Koole W. . A Polymerase Theta-dependent repair pathway suppresses extensive genomic instability at endogenous G4 DNA sites. Nat. Commun. 5, 3216 (2014). 24496117 10.1038/ncomms4216

[b33] Ceccaldi R. . Homologous-recombination-deficient tumours are dependent on Polθ-mediated repair. Nature 518, 258–262 (2015). 25642963 10.1038/nature14184 PMC4415602

[b34] Wood R. D. & Doublie S. DNA polymerase θ (POLQ), double-strand break repair, and cancer. DNA Repair (Amst) 44, 22–32 (2016). 27264557 10.1016/j.dnarep.2016.05.003 PMC5114520

[b35] Saito S., Ura K., Kodama M. & Adachi N. Construction and applications of exon-trapping gene-targeting vectors with a novel strategy for negative selection. BMC Res. Notes 8, 278 (2015). 26123730 10.1186/s13104-015-1241-6 PMC4486125

[b36] Adachi N. . The human pre-B cell line Nalm-6 is highly proficient in gene targeting by homologous recombination. DNA Cell Biol. 25, 19–24 (2006). 16405397 10.1089/dna.2006.25.19

[b37] Iiizumi S. . Simple one-week method to construct gene-targeting vectors: application to production of human knockout cell lines. Biotechniques 41, 311–316 (2006). 16989091 10.2144/000112233

[b38] Suzuki K. . Integration of exogenous DNA into mouse embryonic stem cell chromosomes shows preference into genes and frequent modification at junctions. Chromosome Res. 18, 191–201 (2010). 20177773 10.1007/s10577-010-9111-5

[b39] Roerink S. F., van Schendel R. & Tijsterman M. Polymerase theta-mediated end joining of replication-associated DNA breaks in *C. elegans*. Genome Res. 24, 954–962 (2014). 24614976 10.1101/gr.170431.113 PMC4032859

[b40] Beagan K. & McVey M. Linking DNA polymerase theta structure and function in health and disease. Cell Mol. Life Sci. 73, 603–615 (2016). 26514729 10.1007/s00018-015-2078-9 PMC4715478

[b41] Adachi N., Suzuki H., Iiizumi S. & Koyama H. Hypersensitivity of nonhomologous DNA end-joining mutants to VP-16 and ICRF-193: implications for the repair of topoisomerase II-mediated DNA damage. J. Biol. Chem. 278, 35897–35902 (2003). 12842886 10.1074/jbc.M306500200

[b42] Mali P. . RNA-guided human genome engineering via Cas9. Science 339, 823–826 (2013). 23287722 10.1126/science.1232033 PMC3712628

[b43] Chan S. H., Yu A. M. & McVey M. Dual roles for DNA polymerase theta in alternative end-joining repair of double-strand breaks in Drosophila. PLoS Genet. 6, e1001005 (2010). 20617203 10.1371/journal.pgen.1001005 PMC2895639

[b44] Kawamura K. . DNA polymerase theta is preferentially expressed in lymphoid tissues and upregulated in human cancers. Int. J. Cancer 109, 9–16 (2004). 14735462 10.1002/ijc.11666

[b45] Higgins G. S. . Overexpression of POLQ confers a poor prognosis in early breast cancer patients. Oncotarget 1, 175–184 (2010). 20700469 10.18632/oncotarget.124 PMC2917771

[b46] Lange S. S., Takata K. & Wood R. D. DNA polymerases and cancer. Nat. Rev. Cancer 11, 96–110 (2011). 21258395 10.1038/nrc2998 PMC3739438

[b47] Mateos-Gomez P. A. . Mammalian polymerase θ promotes alternative NHEJ and suppresses recombination. Nature 518, 254–257 (2015). 25642960 10.1038/nature14157 PMC4718306

[b48] Killock D. Targeted therapies: DNA polymerase θ-a new target for synthetic lethality? Nat. Rev. Clin. Oncol. 12, 125 (2015). 25687907 10.1038/nrclinonc.2015.23

[b49] Zelensky A. N., Schimmel J., Kool H., Kanaar R. & Tijsterman M. Inactivation of Polymerase θ and C-NHEJ eliminates off-target integration of exogenous DNA. Nat. Commun. (in the press). 10.1038/s41467-017-00124-3 PMC5501794 28687761

[b50] Hendel A., Fine E. J., Bao G. & Porteus M. H. Quantifying on- and off-target genome editing. Trends Biotechnol. 33, 132–140 (2015). 25595557 10.1016/j.tibtech.2014.12.001 PMC4308725

[b51] Ahrabi S. . A role for human homologous recombination factors in suppressing microhomology-mediated end joining. Nucleic Acids Res. 44, 5743–5757 (2016). 27131361 10.1093/nar/gkw326 PMC4937322

[b52] Wyatt D. W. . Essential roles for polymerase θ-mediated end joining in the repair of chromosome breaks. Mol. Cell 63, 662–673 (2016). 27453047 10.1016/j.molcel.2016.06.020 PMC4992412

[b53] Kent T., Chandramouly G., McDevitt S. M., Ozdemir A. Y. & Pomerantz R. T. Mechanism of microhomology-mediated end-joining promoted by human DNA polymerase θ. Nat. Struct. Mol. Biol. 22, 230–237 (2015). 25643323 10.1038/nsmb.2961 PMC4351179

[b54] Kataoka M. . Alu-mediated nonallelic homologous and nonhomologous recombination in the BMPR2 gene in heritable pulmonary arterial hypertension. Genet. Med. 15, 941–947 (2013). 23579436 10.1038/gim.2013.41

[b55] Higgs D. R. The molecular basis of α-thalassemia. Cold Spring Harb. Perspect. Med. 3, a011718 (2013). 23284078 10.1101/cshperspect.a011718 PMC3530043

[b56] Gu S. . Alu-mediated diverse and complex pathogenic copy-number variants within human chromosome 17 at p13.3. Hum. Mol. Genet. 24, 4061–4077 (2015). 25908615 10.1093/hmg/ddv146 PMC4476451

[b57] Adachi N., Kurosawa A. & Koyama H. Highly proficient gene targeting by homologous recombination in the human pre-B cell line Nalm-6. Methods Mol. Biol. 435, 17–29 (2008). 18370065 10.1007/978-1-59745-232-8_2

[b58] So S., Adachi N., Lieber M. R. & Koyama H. Genetic interactions between BLM and DNA ligase IV in human cells. J. Biol. Chem. 279, 55433–55442 (2004). 15509577 10.1074/jbc.M409827200

[b59] Hurwitz R. . Characterization of a leukemic cell line of the pre-B phenotype. Int. J. Cancer 23, 174–180 (1979). 83966 10.1002/ijc.2910230206

[b60] Wlodarska I. . A new subtype of pre-B acute lymphoblastic leukemia with t(5;12)(q31q33;p12), molecularly and cytogenetically distinct from t(5;12) in chronic myelomonocytic leukemia. Blood 89, 1716–1722 (1997). 9057655

[b61] Liu S. . C-Terminal region of DNA ligase IV drives XRCC4/DNA ligase IV complex to chromatin. Biochem. Biophys. Res. Commun. 439, 173–178 (2013). 23994631 10.1016/j.bbrc.2013.08.068

[b62] Soriano P., Friedrich G. & Lawinger P. Promoter interactions in retrovirus vectors introduced into fibroblasts and embryonic stem cells. J. Virol. 65, 2314–2319 (1991). 2016762 10.1128/jvi.65.5.2314-2319.1991 PMC240581

