# Supplementary material for: Dual loss of human POLQ and LIG4 abolishes random integration
Source: Nat Commun. 2017 Jul 11;8:16112. doi: 10.1038/ncomms16112 (PMC5508229; doi:10.1038/ncomms16112)
Supplement: Supplementary Information [file ncomms16112-s1.pdf]

File name: Supplementary Information
Description: Supplementary figures.

File name: Supplementary Data 1
Description: Junction analysis of *LIG4*-independent RI clones. (a) Junction sequences in RI clones from *LIG4*-/- cells. Genome and vector sequences deleted after the RI event are shown in grey and faint blue, respectively. Inserted nucleotides are indicated in green. The sequence surrounded by a square is identical to a sequence surrounded by a dotted-line square of the same color. MH, microhomology; TI, templated insertion. (b) Summary of junction features of RI clones from *LIG4*-/- cells. ND, not determined.

File name: Supplementary Data 2
Description: Junction analysis of RI clones from *POLQ*-/- cells. (a) Junction sequences in RI clones from *POLQ*-/- cells. Data are presented as in Supplementary Data 1. MH, microhomology; TI, templated insertion; 0-1, 0-1 bp homology. (b) Summary of junction features of RI clones from *POLQ*-/- cells. ND, not determined.

File name: Supplementary Data 3
Description: Junction analysis of RI clones from wild-type cells. (a) Junction sequences in RI clones from wild-type cells. (b) Summary of junction features of RI clones from wild-type cells. Data are presented as in Supplementary Data 2.

File name: Supplementary Data 4
Description: Summary of Cas9-induced DSB joining at the *HPRT* locus. (a-d) Junction sequences in recombinants obtained from wild-type cells (a), *LIG4*-/- cells (b), *LIG4*-complemented *LIG4*-/-*POLQ*-/- cells (#3) (c), and *POLQ*-/- cells (d). Sequences that present 1-bp or ≥2-bp microhomology are shown in pink or light purple, respectively, and indicated at the 5’ flanking sequence. Sequences showing ≥6-bp direct or inverted repeats, which are the hallmarks of templated insertion (TI), are shown in green and underlined. Red denotes mutated bases. (e) Summary of chromosomal DSB joining assay.

File name: Peer review file
Description:

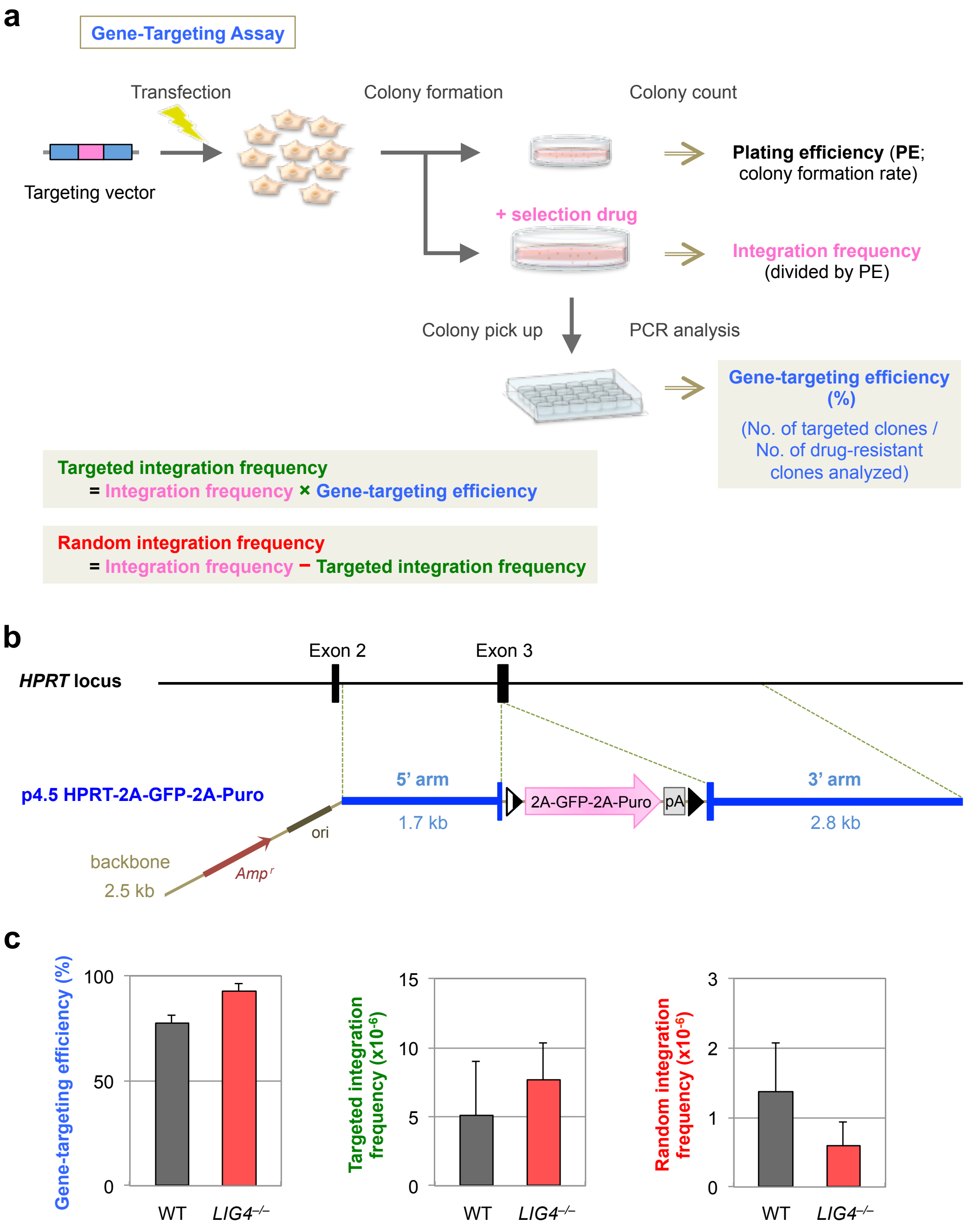

Supplementary Figure 1 | Genetic evidence for a Lig4-independent route of RI. **(a)** Schematic of gene-targeting assay in Nalm-6 cells to calculate gene-targeting efficiency, targeted integration frequency, and RI frequency. **(b)** Structure of the p4.5 HPRT-2A-GFP-2A-Puro vector. **(c)** Gene-targeting efficiency, targeted integration frequency, and RI frequency of p4.5 HPRT-2A-GFP-2A-Puro in wild-type and *LIG4-/-* cells (mean ± s.d.; n=6).

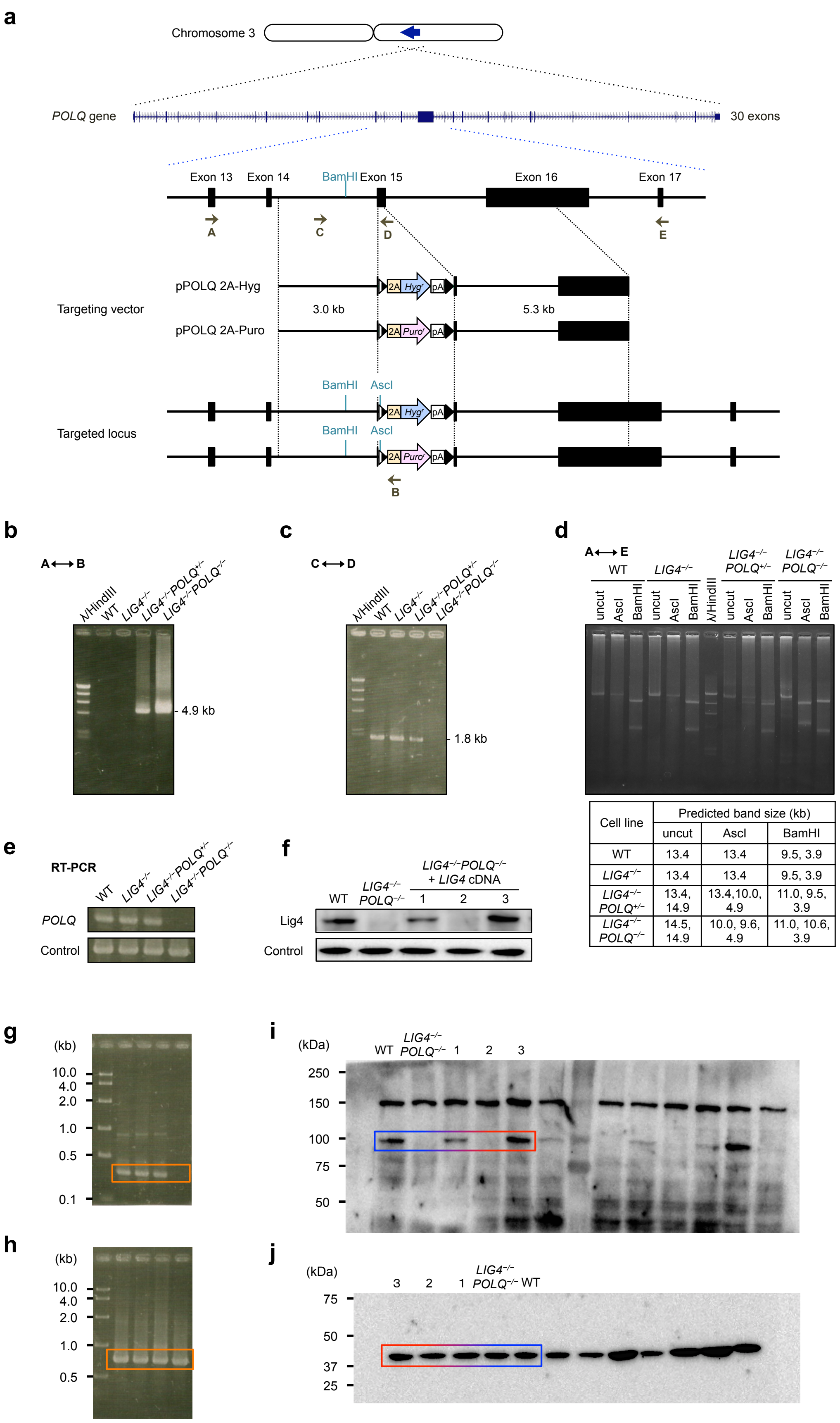

Cell line	Predicted band size (kb)		
	uncut	AscI	BamHI
WT	13.4	13.4	9.5, 3.9
LIG4−/−	13.4	13.4	9.5, 3.9
LIG4−/− *POLQ*+/−	13.4, 14.9	13.4,10.0, 4.9	11.0, 9.5, 3.9
LIG4−/− *POLQ*−/−	14.5, 14.9	10.0, 9.6, 4.9	11.0, 10.6, 3.9

k

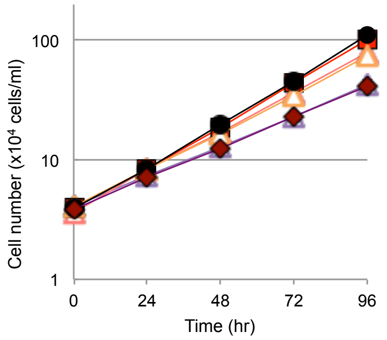

Symbol	Genotype	Doubling time (hr)
●	Wild-type	19.4 ± 0.1
■	*LIG4*$^{-/-}$	19.9 ± 0.3
◆	*LIG4*$^{-/-}$*POLQ*$^{-/-}$	29.0 ± 0.4
△	*LIG4*$^{-/-}$*POLQ*$^{-/-}$ + *LIG4* #1	23.3 ± 0.7
△	*LIG4*$^{-/-}$*POLQ*$^{-/-}$ + *LIG4* #2	29.1 ± 1.3
△	*LIG4*$^{-/-}$*POLQ*$^{-/-}$ + *LIG4* #3	22.7 ± 0.3

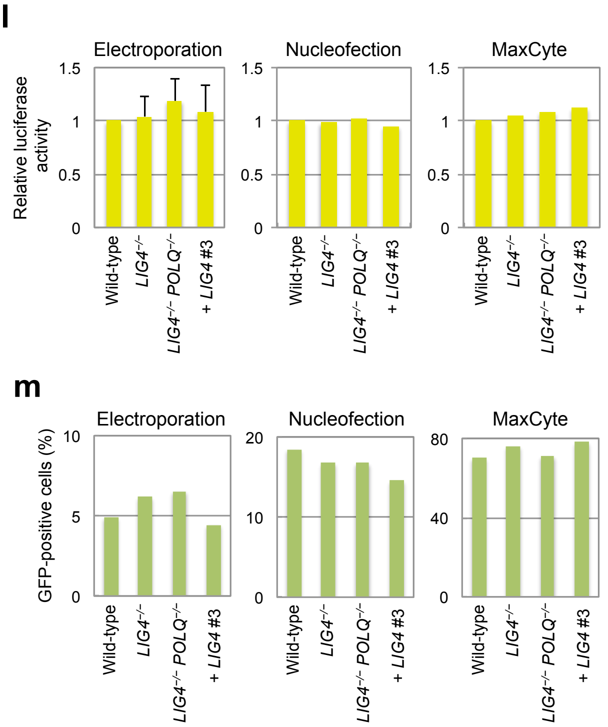

Supplementary Figure 2 | Creation and characteristics of *POLQ*-knockout cell lines. **(a)** Strategy for *POLQ* gene targeting. The human *POLQ* gene is located on the long arm of chromosome 3 and contains 30 exons. 2A, 2A peptide sequence; *Hyg*^{r}, hygromycin-resistance gene; *Puro*^{r}, puromycin-resistance gene. **(b)** PCR analysis to confirm the correct gene targeting at the 5' side. Primers used (A and B) are shown in panel **a**. WT, wild-type. **(c)** PCR analysis to confirm the disruption of exon 15 of *POLQ*. Primers used (C and D) are shown in panel **a**. **(d)** PCR analysis to confirm the gene knockout via HR-mediated gene targeting. Primers used (A and E) are shown in panel **a**. The PCR products were further subjected to restriction enzyme digestion to confirm the sizes of DNA bands, which are shown in the lower panel. **(e)** RT-PCR analysis to confirm the absence of *POLQ* expression. Primers used (A and D) to amplify a *POLQ* cDNA (251 bp) from untargeted cells are shown in panel **a**. **(f)** Western blot analysis for Lig4. Note that ectopic expression of *LIG4* cDNA in *LIG4-/-POLQ-/-* cells was successful in clones #1 and #3, but not clone #2. **(g,h)** Uncropped agarose gels for panel **e**. **(i,j)** Uncropped blots for panel **f**. **(k)** Growth curves of the indicated cell lines (mean ± s.d.; n=3). **(l)** Transient expression assay using a luciferase expression vector. Transfection of pCMV-Nluc was performed by the indicated methods, and luciferase activity was compared between the cell lines. Data are the mean ± s.d. of three independent experiments (left). **(m)** Transient expression assay using a GFP expression vector. Transfection of pmaxGFP was performed by the indicated methods, and the percentage of GFP-positive cells was compared between the cell lines.

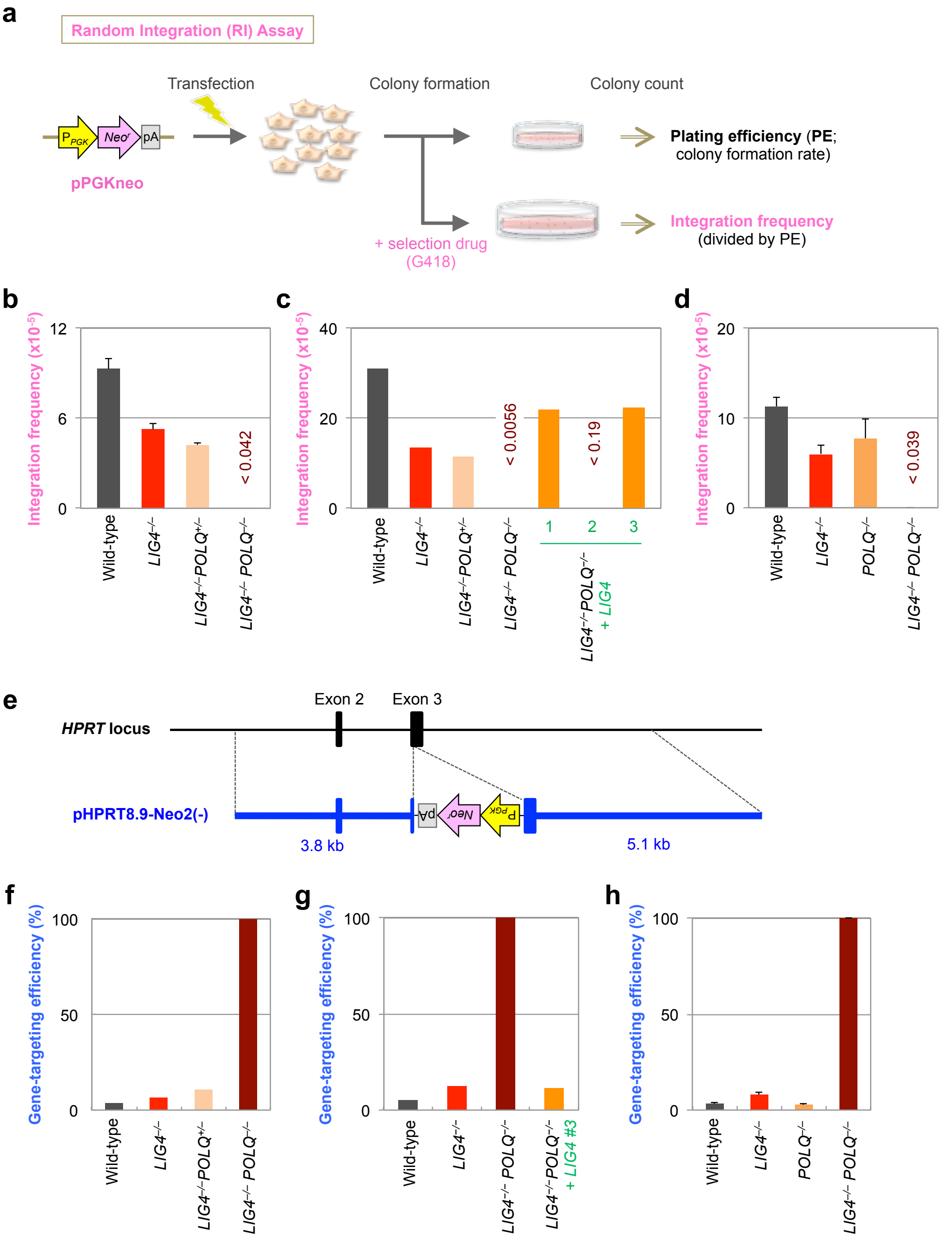

Supplementary Figure 3 | Confirmation of RI suppression by the loss of Pol θ and Lig4. **(a)** Schematic of RI assay. P_{PGK}, PGK promoter; *Neo*^{r}, neomycin-resistance gene. **(b)** RI frequency in the indicated cell lines electroporated with pPGKneo (mean ± s.d.; n=3). **(c)** RI frequency in the indicated cell lines Nucleofected with pPGKneo. **(d)** RI frequency in the indicated cell lines electroporated with pPGKneo (mean ± s.d.; n=3). **(e)** Structure of pHPRT8.9-Neo2(-), a promoter-containing *HPRT* targeting vector. Symbols are as in **a**. **(f)** Gene-targeting efficiency of pHPRT8.9-Neo2(-) in the indicated cell lines. Data are the mean of two independent experiments. **(g)** Gene-targeting efficiency in the indicated cell lines Nucleofected with pHPRT8.9-Neo2(-). **(h)** Gene-targeting efficiency in the indicated cell lines electroporated with pHPRT8.9-Neo2(-) (mean ± s.d.; n=3).

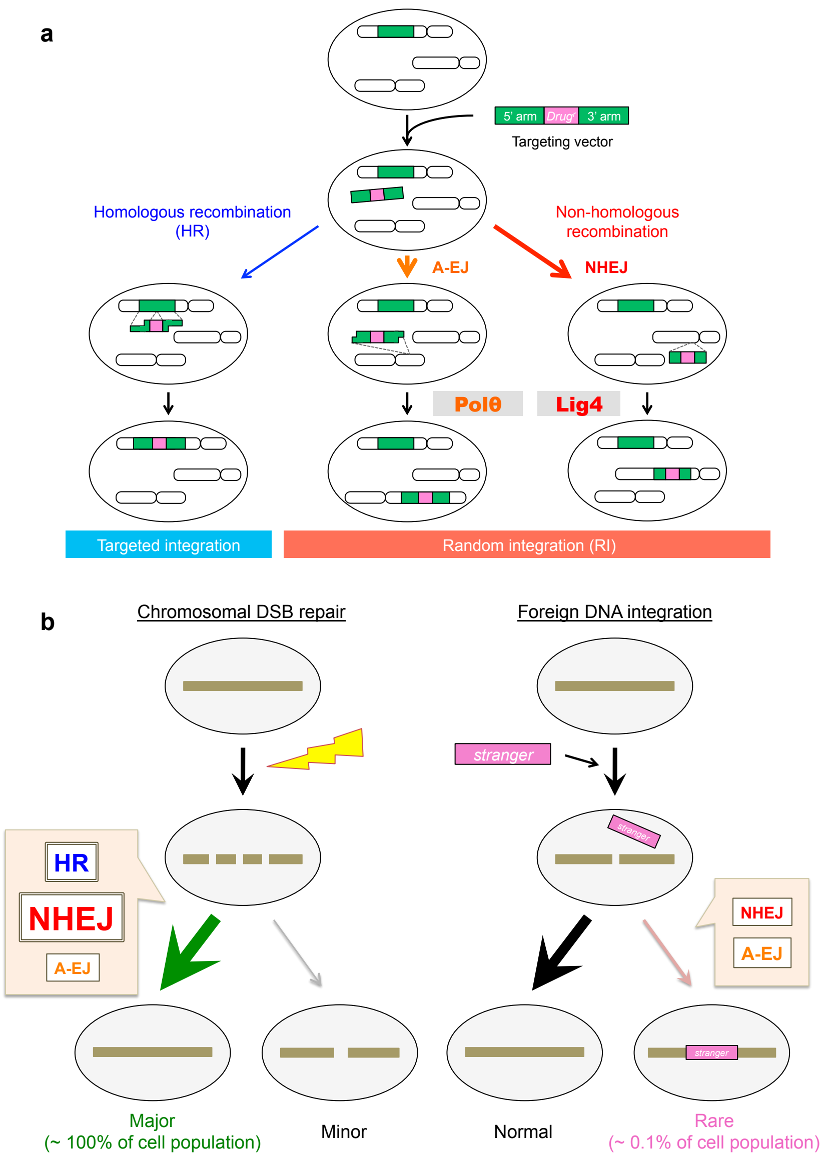

Supplementary Figure 4 | Differential roles of human DSB repair pathways. **(a)** Schematic representation of homologous and non-homologous integration of targeting vector. Lig4 and Pol θ are indispensable for NHEJ and A-EJ, respectively. Dual loss of these two proteins suppresses RI, thereby only allowing targeted integration. **(b)** Distinct contributions of NHEJ and A-EJ in chromosomal DSB repair and foreign DNA integration. Upon DSBs, cells need repair to survive or preserve genome integrity, and thus try to fix them basically (left panel). By contrast, upon foreign DNA transfection, cells do not need to incorporate the "*stranger*" and perhaps try not to use it as a recombination substrate. NHEJ plays a major role in DSB repair, but its absence has little or no impact on RI. A-EJ only has a minor role in DSB repair, but makes a significant contribution to RI. Although not shown in the figure, the absence of both NHEJ and A-EJ uncovers rare recombination events that utilize a stretch of homology longer than that seen in A-EJ.
